# A multidisciplinary cognitive behavioural programme for coping with chronic neuropathic pain following spinal cord injury: the protocol of the CONECSI trial

**DOI:** 10.1186/1471-2377-10-96

**Published:** 2010-10-20

**Authors:** Matagne Heutink, Marcel WM Post, Peter Luthart, Lilian EMA Pfennings, Catja A Dijkstra, Eline Lindeman

**Affiliations:** 1 Rudolf Magnus Institute of Neuroscience and Center of Excellence for Rehabilitation Medicine, University Medical Center Utrecht and Rehabilitation Center De Hoogstraat, Utrecht, The Netherlands; 2 Department of Spinal Cord Management, Rehabilitation Center De Hoogstraat, Utrecht, The Netherlands

## Abstract

**Background:**

Most people with a spinal cord injury rate neuropathic pain as one of the most difficult problems to manage and there are no medical treatments that provide satisfactory pain relief in most people. Furthermore, psychosocial factors have been considered in the maintenance and aggravation of neuropathic spinal cord injury pain. Psychological interventions to support people with spinal cord injury to deal with neuropathic pain, however, are sparse. The primary aim of the CONECSI (COping with NEuropathiC Spinal cord Injury pain) trial is to evaluate the effects of a multidisciplinary cognitive behavioural treatment programme on pain intensity and pain-related disability, and secondary on mood, participation in activities, and life satisfaction.

**Methods/Design:**

CONECSI is a multicentre randomised controlled trial. A sample of 60 persons with chronic neuropathic spinal cord injury pain will be recruited from four rehabilitation centres and randomised to an intervention group or a waiting list control group. The control group will be invited for the programme six months after the intervention group. Main inclusion criteria are: having chronic (> 6 months) neuropathic spinal cord injury pain as the worst pain complaint and rating the pain intensity in the last week as 40 or more on a 0-100 scale. The intervention consists of educational, cognitive, and behavioural elements and encompasses 11 sessions over a 3-month period. Each meeting will be supervised by a local psychologist and physical therapist. Measurements will be perfomed before starting the programme/entering the control group, and at 3, 6, 9, and 12 months. Primary outcomes are pain intensity and pain-related disability (Chronic Pain Grade questionnaire). Secondary outcomes are mood (Hospital Anxiety and Depression Scale), participation in activities (Utrecht Activities List), and life satisfaction (Life Satisfaction Questionnaire). Pain coping and pain cognitions will be assessed with three questionnaires (Coping Strategy Questionnaire, Pain Coping Inventory, and Pain Cognition List).

**Discussion:**

The CONECSI trial will reveal the effects of a multidisciplinary cognitive behavioural programme for people with chronic neuropathic spinal cord injury pain. This intervention is expected to contribute to the rehabilitation treatment possibilities for this population.

**Trial Registration:**

Dutch Trial Register NTR1580.

## Background

Around 65-85% of the people with spinal cord injury (SCI) experience persistent pain and around one-third of these suffer from severe pain [1]. People with SCI consistently rate chronic SCI pain (CSCIP) as one of the most difficult problems to manage, despite the presence of other problems that interfere with daily life [2], and it is a major impediment to effective rehabilitation [3]. Several types of pain may occur following SCI. In the taxonomy of The Spinal Cord Injury Pain Task Force of the International Association of the Study of Pain, pain types are divided into nociceptive (musculoskeletal or visceral) and neuropathic (above-level, at-level, or below-level) pain [4]. Neuropathic pain is initiated by a primary injury to the nervous system and involves abnormal sensations, such as burning, electric and shooting, and often reduced touch sensation and allodynia [4]. About one third of all people with SCI develop below-level neuropathic pain and this pain type is the most likely type of SCI pain to be described as severe or excruciating [5].

Our understanding of the mechanisms underlying chronic neuropathic SCI pain (CNSCIP) is still incomplete [1,6,7] and, consequently, treatment often is a matter of trial-and-error [8]. Although two recent pharmacological trials showed some success in alleviating neuropathic pain [9,10], none of the treatments that are currently available (e.g., pharmacological treatment, physical methods, surgical interventions) relieve pain in the majority of the SCI population [1,6,11].

The biopsychosocial view provides an integrated model that incorporates mechanical and physiological processes as well as psychological and social/contextual variables that may cause and perpetuate chronic pain [12]. Strong relationships between psychosocial factors and SCI-related pain have been found [13,14]. Results of several studies indicate that pain cognitions like catastrophizing [13,15-18] and pain coping strategies like passive coping [13,17] were significant predictors of pain intensity and pain-related disability. Psychosocial factors were even stronger associated with the experience of pain than physiological factors were [19]. These findings suggest that psychological interventions may be useful to improve the quality of life of persons suffering from CNSCIP.

Cognitive behavioural therapy (CBT) aimed at modifying dysfunctional pain cognitions and coping abilities, has been shown to be effective in people with low back pain [20] and has been successfully applied as a treatment of depression and anxiety in SCI [21]. Interventions like CBT might therefore be successfully applied in SCI rehabilitation as part of a multidisciplinary programme including educational, physical, psychological, and social aspects of pain treatment [22]. Although one small controlled study showed positive effects of a comprehensive CBT programme for people with CNSCIP on mood [8], no other evaluation studies are available, so there is insufficient evidence for the effectiveness of cognitive behavioural interventions as a treatment of CNSCIP to date [23]. In conclusion, there is a need for randomised controlled trials (RCT) for the evaluation of cognitive behavioural interventions targeted at coping with CNSCIP.

### Aims of the study

The primary aim of the CONECSI (COping with NEuropathiC Spinal cord Injury pain) trial is to evaluate the effectiveness of a multidisciplinary cognitive behavioural programme for coping with CNSCIP. The intervention is expected to result in decreased pain intensity and pain-related disability, and in higher levels of mood, participation in activities, and life satisfaction. The secondary aim is to examine post-hoc (explorative) the influence of demographic and lesion characteristics, pain coping and pain cognitions on the effect of the intervention and the relation between the change of coping and cognitions and the effectiveness of the intervention. It is expected that the intervention will be most effective in persons who show dysfunctional pain cognitions and pain coping strategies before the intervention and in persons who show a change in pain coping and pain cognitions in a positive direction. The third aim is to examine the satisfaction of the participants and trainers about the intervention and to explore which parts will be regarded as effective. This paper describes the design of the CONECSI trial and the content of the programme.

## Methods/Design

### Study design

A multicentre RCT will be conducted to evaluate the effects of the multidisciplinary cognitive behavioural programme for coping with CNSCIP in four Dutch rehabilitation centres. Within each participating rehabilitation centre, participants will be randomly allocated to the intervention group or to the waiting list control group. The control group will be invited for the programme after a waiting period of six months.

### Ethical considerations

The Medical Ethics Committee of the University Medical Centre Utrecht and the participating rehabilitation centres have approved the study protocol. Written informed consent will be obtained from each participant. The trial is registered in the Dutch Trial Register (NTR1580).

### Setting

The study will be conducted in four rehabilitation centres with a specialisation in SCI rehabilitation in different parts of the Netherlands: 'De Hoogstraat' in Utrecht; 'Het Roessingh' in Enschede; 'Rijndam' in Rotterdam, and 'Adelante zorggroep' in Hoensbroek.

### Participants and procedure

**Figure 1:**
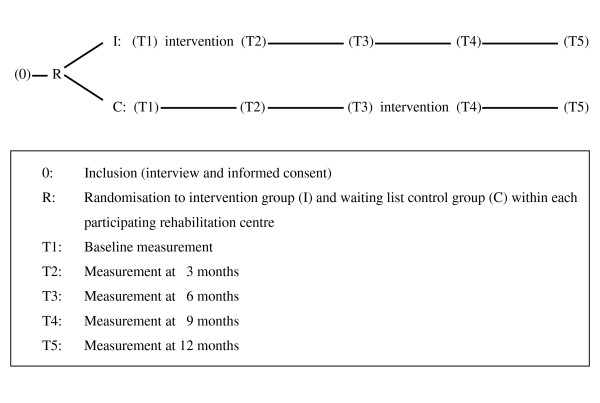
**Flow chart**.

Physiatrists from the four rehabilitation centres select former patients from their centre meeting inclusion criteria 1, 2, and 3 (Table 1). The selected persons will be sent a questionnaire to determine if they meet the inclusion (4, 5, and 6) and exclusion (1) criteria. In an interview with the psychologist, who will conduct the programme, will be checked for the other exclusion criteria and will be asked for written consent before final inclusion in the CONECSI trial. Included participants will be randomised (stratified by rehabilitation centre) to the intervention group or the waiting list control group (Figure 1). Measurements will be performed in both groups before starting the programme or entering the control group (t1), at 3 (t2), 6 (t3), 9 (t4), and 12 months (t5) follow-up. The control group will be invited for the programme after the follow-up measurement at 6 months.

**Table 1:** In-and exclusion criteria

Inclusion criteria
(1) SCI

(2) at least 18 years old

(3) at least one year after discharge from first inpatient SCI rehabilitation

(4) main pain type is neuropathic pain

(5) duration of neuropathic pain of at least six months

(6) pain intensity score in the previous week of at least 40 on the 0-100 numerical rating scale of the Chronic Pain Grade

**Exclusion criteria**

(1) SCI caused by metastatic tumour

(2) previous cognitive behavioural therapy for coping with pain after SCI

(3) inability to function in a group because of psychopathology

(4) insufficient mastery of the Dutch language

### Intervention

This multidisciplinary programme is developed for people with CNSCIP. The programme consists of ten sessions of three hours over a ten-week period and a comeback session three weeks after the tenth session. Each meeting will be supervised by a psychologist and physiotherapist (the trainers) from the local centre. There will be a maximum of ten participants per group. The programme comprises educational, cognitive, and behavioural elements targeted at coping with CNSCIP. At the first session, participants will receive a course book containing information on all sessions, reading texts, and homework assignments. All participants will have a buddy who will attend two sessions. The buddy is the partner, family member, or a good friend of the participant. The buddy will be asked to read the course material, to help (if necessary) with the homework assignments, and the participants can discuss the intervention with the buddy. The trainers will receive the same course book as the participants, but with an extended protocol for each session. Additional file 1: Appendix 1 gives an overview of the main contents of the 11 sessions.

#### Psycho-education

Two theoretical models will be used in the programme: the BioPsychoSocial (BPS) model [24] and the Activating event-Belief-Consequence (ABC) model [25]. These two models will be explained in educational sessions and in guided group discussions using fictitious cases. These models will also be applied in sports workshops and homework assignments.

#### BPS model

According to the BPS model [24] biological, psychological, and social factors contribute to the experience of pain [6]. The model will be used to focus on the influence of psychosocial factors (e.g., beliefs, relationships, stress) on the experience of pain, to clarify the relationship between biological, psychological, and social factors and pain, and to clarify the importance of maintaining a balance between capacity and load.

#### ABC model

Rational Emotive Behaviour Therapy (REBT) [25] proposes a 'biopsychosocial' explanation of causation, i.e., a combination of biological, psychological, and social factors are involved in the way humans feel and behave. The ABC model, an element of REBT, is used for cognitive restructuring. The ABC model is based on the fundament that most cases people do not merely get upset by adversities or situations, but also by their views and thoughts, beliefs, attitudes and self-efficacy expectations, about the world, themselves, and others. The model states that it normally is not only an activating event (A) that contributes to disturbed and dysfunctional emotional and behavioural consequences (C), but also what people believe (B) about the activating event (A). In this programme the ABC model will be used to teach participants to become aware of irrational beliefs, dysfunctional pain cognitions, and maladaptive coping, and they will be encouraged to change these thoughts and behaviours. Therefore, the trainers show the participants how to find and dispute (D) their irrational beliefs and dysfunctional pain cognitions and formulate new functional cognitions and rational coping beliefs. And subsequently judge the effect (E, arrive effective new philosophies or rational coping beliefs) of this new cognitions on emotional and behavioural consequences [26].

Further, the trainers will be assisted by two physiatrists and a role model in three sessions (Appendix 1). A local physiatrist specialised in SCI rehabilitation will provide information about SCI, pain physiology and pain classification, pharmacological and non-pharmacological SCI pain treatments, and their limitations. A local physiatrist specialised in chronic pain rehabilitation will provide information about chronic pain and chronic pain rehabilitation for other pain diagnoses than SCI. The role model will tell his/her story of living with CNSCIP and how he/she was able to live his/her life despite the pain.

#### Workshops

Four sessions will include a workshop on relaxation exercises with attention to terms for relaxation, breathing and body sensations (Appendix 1). The exercises target on stress reduction, restore balance, attention shift and awareness of available energy. Three sessions will include sports workshops in which exercises in circuit (power) training will be performed (Appendix 1). After these workshops the participants will take a closer look at their cognitions during the workshops (e.g., 'I have to work out the best I can' or 'I'm stupid because I'm not good at sports anymore') with the ABC model and at their balance between capacity and load with the BPS model.

### Outcome measures

Primary outcome measure will be pain intensity and pain-related disability. Secondary outcome measures will be mood, participation in activities, and life satisfaction. All outcome measures and instrumentation are presented in Table 2.

**Table 2:** Outcome measures and instrumentation

Outcome measures	Instrumentation	T1	T2	T3	T4	T5
**Primary outcome measures**						

Pain intensity and Pain-related disability	Chronic Pain Grade questionnaire	X	X	X	X	X

**Secondary outcome measures**						

Mood	Hospital Anxiety and Depression Scale	X	X	X	X	X
Participation in activities	Utrecht Activities List	X	X	X	X	X
Life satisfaction	Life Satisfaction Questionnaire	X	X	X	X	X

**Pain coping and pain cognitions**						

Pain coping	Coping Strategy Questionnaire	X	X	X^I^		X^C^
	Pain Coping Inventory	X	X	X^I^		X^C^
Pain cognitions	Pain Cognition List	X	X	X^I^		X^C^

**Other measures**						

Demographic variables (age, gender, marital status, and education)		X				

SCI and pain characteristics		X				

Neuropathic pain treatment		X	X	X	X	X

Functional independence	Barthel Index	X				

Satisfaction with intervention	Questionnaire on satisfaction		X^I^		X^C^	

X: intenvention group and waiting list control group

X^I^:only intervention group

X^C^: only waiting list control group

#### Chronic Pain Grade questionnaire (CPG)

The CPG [27] is used to assess pain intensity and pain-related disability. Participants have to rate their pain intensity on a 0-10 Numeric Rating Scale (NRS) for average pain, worst pain, and current pain (pain intensity score), and the degree of pain interference with daily activities, work/household activities, and recreational/social activities (pain-related disability score). The internal consistency for the pain intensity score and the pain-related disability score in an SCI population is excellent (Cronbach's alpha 0.95 and 0.94, respectively) [13]. In this study, the CPG is slightly adjusted to ask for neuropathic pain in the past week instead of the past six months.

#### Hospital Anxiety and Depression Scale (HADS)

The HADS is a 14-item self-report measure, which is used for scoring mood. It contains two 7-item scales: one for anxiety and one for depression, both with a score range of 0-21. It is a valid and reliable measure and responsive to change [28]. Woolrich and colleagues report a good internal consistency within an outpatient population with SCI [29]: the Cronbach's alpha for the anxiety scale is 0.85 and for the depression scale 0.79.

#### Utrecht Activities List (UAL)

The UAL [30,31] is a Dutch adaptation of the Craig Handicap Assessment and Rating Technique (CHART) [32] and is used to assess participation in activities. The questionnaire assesses the time spent on activities such as paid work, study, housekeeping, voluntary work, hobbies, and sports in hours per week, and the number of contacts per week with family, friends and acquaintances, and neighbours.

#### Life Satisfaction Questionnaire (LiSat-9)

The LiSat-9 [33] is used to assess life satisfaction and consists of a global item 'life as a whole' and eight domain-specific items 'activities of daily living', 'leisure', 'vocational situation', 'financial situation', 'sexual life', 'partnership relationship', 'family life', and 'contacts with friends'. These nine variables are rated on a six point scale (very dissatisfying to very satisfying), with higher scores reflecting greater satisfaction. The internal consistency of the total score (average of all item scores) is good (Cronbach's alpha of 0.80) in a Dutch SCI population [34].

### Pain coping and pain cognitions

#### Coping Strategy Questionnaire (CSQ)

The Dutch version of the CSQ [35] is an adaptation of the CSQ developed by Rosenstiel and Keefe [36]. The main difference between the original CSQ and the Dutch adaptation is a different answering format. In the Dutch adaptation people mark 10 cm visual analogue scales with the end-points defined in the same way as on the original seven-point Likert scale, i.e., as 'never do' and 'always do' [35]. The CSQ consists of 44 items in eight subscales to assess the use of six different cognitive strategies ('Diverting Attention', 'Re-interpreting Pain Sensations', 'Coping Self-Statements', 'Ignoring Pain Sensations', 'Catastrophizing', and 'Praying/Hoping'), and one behavioural strategy ('Increasing Activity Level'). Participants also rate how effective they think they are in controlling and decreasing pain ('Perceived Effectiveness'). A high score on a particular subscale indicates a higher endorsement of the cognitive coping strategy. The internal consistency is satisfactory to good (Cronbach's alpha range from 0.67 to 0.81) and the stability is reasonable to good (stability coefficients with a time interval of eight weeks range from 0.45 to 0.86) [37].

#### Pain Coping Inventory (PCI)

The PCI consists of 33 items with a four-point Likert scale, from 1 (rarely or never) to 4 (very often). The following six scales are distinguished, divided in active pain coping dimensions: 'Pain Transformation', 'Distraction', 'Reducing Demands', and passive pain coping dimensions: 'Retreating', 'Worrying', and 'Resting', all of which are internally reliable [38]. The internal consistency for the six scales range from 0.62 to 0.79 [39].

#### Pain Cognition List (PCL-2003)

The PCL-2003 consists of 39 items. Each item presents a specific pain cognition statement, for example, 'My thoughts are always concentrated on the pain'. Items are scored on a five-point Likert scale, from 1 (totally disagree) to 5 (totally agree) [40]. The questionnaire consists of five subscales ('Catastrophizing', 'Restrictions', 'Optimism', 'Internal Control', and 'Reliance on health care'), with varying length (from 4 to 16 items) and internal consistency (Cronbach's alpha of .64 to .88 with an average of 0.75), and with correlations between subscales ranging from .00 to .45. Validity of these subscales is supported by the meaningful pattern of correlations with other relevant constructs [41]. The stability of the PCL-2003 is satisfactory with the Pearson correlation coefficients with a time interval of two weeks ranging between 0.51 ('Reliance on health care') and 0.73 ('Catastrophizing') with an average of 0.64 [41].

Two questionnaires will be used for assessing pain coping, because they cover partly different coping styles and are additional to each other. The PCI assesses passive coping styles (like 'Resting' and 'Retreating', for example 'Slowing down when in pain'), while the CSQ assesses active coping styles (like 'Increasing Activity Level', for example 'I leave my home going to do something, like going to the movies or go shopping') and other coping styles that the PCI does not assess ('Ignoring Pain Sensations', 'Coping Self-Statements', and 'Praying/Hoping'). There is an overlap in the subscale 'Catastrophizing' between the CSQ, PCI, and PCL, but the PCL assesses people's cognitions (like 'I think fate has hit me') instead of coping ('When I'm in pain, I worry all the time about whether the pain will end').

### Compliance

In order to conduct this trial in a uniform way in the four rehabilitation centres, a detailed course book and protocol for each session have been written and a one-day training for the trainers will be organized. One of the authors (MH) will monitor on a regular basis (attending two sessions of the intervention group and two sessions of the control group in each centre) whether the content of the intervention will be executed as intended in the four rehabilitation centres. Compliance will be assessed by recording the number of sessions attended.

Neuropathic pain treatments will be registered at all measurements by using a pre-coded list including common pharmacological treatments, physical methods (e.g., TENS), surgical interventions, psychological treatment, alternative treatment (e.g., acupuncture), and other treatments. Participants will be allowed to continue their current pain treatments, but will be asked to refrain, if possible, from starting, stopping, or changing pain treatment during the intervention.

### Power analysis

The power analysis is based on information from a previous cross-sectional study [13], in which a mean pain intensity score on the CPG of 64.7 (SD 17.0) was seen in the subgroup of patients with a pain intensity score of 40 or higher. A mean difference of 16 points (corresponding to a 25% reduction from score 65 to 49) on the pain intensity score of the CPG between the intervention group and the control group in favour of the intervention group is regarded as clinically relevant. A minimum of 24 persons is required for each arm of the trial (total of 48 persons) to achieve sufficient statistical power (alpha 0.05, beta 0.90, one-tailed test). Expecting a maximum dropout rate of 20%, we aim to include 60 persons.

### Data analysis

This trial with repeated measurements nested in each patient will be used to evaluate differences in effect between the intervention group and the waiting list control group. Differences between groups, with 95% confidence intervals, will be calculated for the outcome measures: CPG, HADS, UAL, and LiSat-9, by random coefficient (multilevel) analyses. The effectiveness of the intervention will be tested by analysing differences between the intervention group and the waiting list control group in the course of outcomes over time (0, 3, and 6 months, respectively t1, t2, and t3). Further, the long-term (6, 9, and 12 months, respectively t3, t4, and t5) impact of the intervention will be analysed in participants randomised to the intervention group. The impact of demographic and lesion characteristics, pain cognitions and pain coping on the effectiveness of the intervention will be studied explorative by merging data from the intervention group (t1, t2, t3) and the waiting list group (t3, t4, t5). Both the impact of differences at baseline, and changes in pain cognitions and pain coping during and after the intervention will be post-hoc analysed. Factor rehabilitation centre will be controlled for in the effectiveness analyses, but post-hoc analyses of differences between rehabilitation centres will be analysed and combined with information on compliance and patient satisfaction to identify determinants of a successful intervention. Data will be analysed according to the intention-to-treat principle. SPSS statistical program for Windows (version 16.0) and the MLwiN program of the Centre for Multilevel Modelling, Institute of Education, University of London (version 2.02) will be used for the analyses. Significance level will be set at a p-value less than .05.

## Discussion

This multidisciplinary cognitive behavioural programme for coping with CNSCIP will be evaluated in a multicentre trial, in which 60 persons will be randomised to an intervention group or a waiting list control group in four participating rehabilitation centres. The CONECSI trial meets the need for an RCT for the evaluation of CBT for people with CNSCIP, focusing on reduction of negative pain cognitions and promoting adaptive coping. This intervention is expected to contribute to the treatment options for people with CNSCIP, a severe problem for which existing treatments are insufficiently effective.

This study will have the advantage of a randomised control group over other studies like Norrbrink Budh and colleagues [8]. Our use of a waiting list control group that will be invited for the intervention six months after the intervention group has potential advantages. First of all, the people who will be randomised in the waiting list group will also be given the opportunity to participate in the intervention. This prospective might minimize demoralization and enhance participation of people in the waiting list group. Second, the possible impact of pain coping and pain cognitions on the effectiveness of the intervention can be examined in twice as many people.

The intervention consists of an eclectic and multidisciplinary treatment programme: a combination of education, relaxation and activation, and using different theoretical models that are complimentary to each other. If the intervention as a whole is effective, this will make it more difficult to point out the effective elements of the intervention. But we expect to include participants with a variety of pain cognition and pain coping behaviours (for example, an active coping over-user and a passive coping, high catastrophizing under-user) and therefore we choose to present information on different topics and in different ways. The expected advantage is that, this way, each participant will find something useful in the programme.

Most people with SCI have several pain types simultaneously [42]. Therefore, including people who exclusively have CNSCIP is not an option and we decided to include people who experience CNSCIP as their main type of pain. The aim of treating persons with CBT is to learn them to cope with CNSCIP and not primarily to lower the intensity of neuropathic pain. But we think this might have an effect on the intensity of pain as well, as was shown in persons with chronic low back pain [20]. Further, we use a pain-related disability score and not a generic disability measure to minimize the influence of the paralysis and other secondary conditions of the disability on outcomes of this trial.

If this new intervention turns out to be effective, its chances on implementation in the rehabilitation setting in the Netherlands are good. First, the multidisciplinary design fits well in a rehabilitation setting. Second, the treatment protocol will already be applied in four rehabilitation centres at that time and their experiences with this intervention will facilitate implementation in other Dutch rehabilitation centres. Third, the availability of a written treatment protocol also contributes to applicability in other rehabilitation centres by rehabilitation professionals. Finally, it is important that application of this type of interventions as part of rehabilitation outpatient treatment is eligible for reimbursement by health insurances in the Netherlands.

## Competing interests

The authors declare that they have no competing interests.

## Authors' contributions

MP developed the idea and procured funding for the study. All authors contributed to the design and the protocol of the study, and content of the programme. All authors reviewed the manuscript and approved the final version.

## Pre-publication history

The pre-publication history for this paper can be accessed here:

http://www.biomedcentral.com/1471-2377/10/96/prepub

## Supplementary Material

Additional file 1 **Appendix 1: Main contents of the 11 sessions**. A table with the 11 sessions of the programme and a summary of the main content of each session. Click here for file
