# Supplementary material for: A multidisciplinary cognitive behavioural programme for coping with chronic neuropathic pain following spinal cord injury: the protocol of the CONECSI trial
Source: BMC Neurol. 2010 Oct 20;10:96. doi: 10.1186/1471-2377-10-96 (PMC2984384; doi:10.1186/1471-2377-10-96)
Supplement: Additional file 1 — Appendix 1: Main contents of the 11 sessions. A table with the 11 sessions of the programme and a summary of the main content of each session. [file 1471-2377-10-96-S1.DOC]

| **Sessions** | **Main contents** |
| --- | --- |
| Session 1 | Education BPS model  Education SCI and CNSCIP  Goal setting |
| Session 2  Buddies | Education ABC model (ABC)  Education by physiatrist specialised in SCI rehabilitation |
| Session 3 | Expansion ABC model (ABCDE) Education by physiatrist specialised in chronic pain rehabilitation  Education movement and pain |
| Session 4 | Sports workshop  Evaluation sports workshop with ABC model |
| Session 5 | Education assertiveness and communication about pain  Education by role model  Introduction relaxation exercises  Evaluation goals |
| Session 6 | Education pain, mood, and stress  Workshop relaxation exercises  Evaluation workshop relaxation exercises |
| Session 7 | Sports workshop  Evaluation sports workshop with BPS model and ABC model |
| Session 8  Buddies | Education social aspects and partner, family, and friends  Workshop relaxation exercises  Evaluation workshop relaxation exercises |
| Session 9 | Sports workshop  Evaluation sports workshop with ABC model  Workshop relaxation exercises  Evaluation workshop relaxation exercises |
| Session 10 | Summary and rehearsal contents intervention  Evaluation goals  Application in daily life |
| Session 11 | Summary contents intervention  Evaluation intervention |
